# New Kid on the Block: The Efficacy of Phytomedicine Extracts Urox^®^ in Reducing Overactive Bladder Symptoms in Rats

**DOI:** 10.3389/fmolb.2022.896624

**Published:** 2022-06-21

**Authors:** Łukasz Zapała, Kajetan Juszczak, Przemysław Adamczyk, Jan Adamowicz, Aleksander Ślusarczyk, Tomasz Kluz, Marcin Misiek, Artur Rogowski, Magdalena Emilia Grzybowska, Klaudia Stangel-Wójcikiewicz, Mikołaj Piotr Zaborowski, Ewa Poleszak, Piotr Radziszewski, Andrzej Wróbel

**Affiliations:** ^1^ Clinic of General, Oncological and Functional Urology, Medical University of Warsaw, Warsaw, Poland; ^2^ Chair of Urology and Andrology, Collegium Medicum in Bydgoszcz, Nicolaus Copernicus University in Torun, Bydgoszcz, Poland; ^3^ Department of General and Oncologic Urology, Nicolaus Copernicus Hospital, Torun, Poland; ^4^ Clinic of General and Oncologic Urology, Collegium Medicum of Nicolaus Copernicus University, Bydgoszcz, Poland; ^5^ Department of Gynecology, Gynecological Oncology and Obstetrics, Institute of Medical Sciences, Medical College of Rzeszow University, Rzeszow, Poland; ^6^ Department of Gynecologic Oncology, Holy Cross Cancer Center, Kielce, Poland; ^7^ Department of Minimally Invasive and Endoscopic Gynecology, Military Institute of Medicine, Legionowo Hospital, Legionowo, Poland; ^8^ Department of Gynecology, Gynecological Oncology and Gynecological Endocrinology, Medical University of Gdansk, Gdansk, Poland; ^9^ Department of Gynecology and Oncology, Jagiellonian University Medical College, Cracow, Poland; ^10^ Department of Gynecology, Obstetrics and Gynecologic Oncology, Division of Gynecologic Oncology, Poznan University of Medical Sciences, Poznań, Poland; ^11^ Laboratory of Preclinical Testing, Chair and Department of Applied and Social Pharmacy, Medical University of Lublin, Lublin, Poland; ^12^ Second Department of Gynecology, Medical University of Lublin, Lublin, Poland

**Keywords:** phytomedicine, overactive bladder, Urox, herbal drugs, biomarkers

## Abstract

The aim of the current study was to determine if phytomedicine (Urox^®^) would reverse retinyl acetate (RA)–induced changes characteristic of bladder overactivity. There were 60 rats divided into the following 4 groups: I—control, II—received RA to induce detrusor overactivity (DO), III—received Urox (840 mg daily for 14 days), and IV—received combination of RA and Urox^®^. The cystometry was performed 2 days after the last dose of Urox^®^. Next, urothelium thickness and biochemical parameter measurements were performed. In group IV, a decrease in basal pressure and detrusor overactivity index was noted when compared to group II. Furthermore, in group IV the following parameters were increased: threshold pressure, voided volume, intercontraction interval, and bladder compliance in comparison with group II. There were significant elevations in c-Fos expression in the neuronal voiding centers in group II, while the expression of c-Fos in group IV was normalized. No significant changes in the values of the analyzed biomarkers in group III were found, while in group II, an elevation in BDNF, NGF, CGRP, ATP, Rho kinase, malondialdehyde, 3-nitrotyrosine, TRPV1, OCT-3, and VAChT and then a decrease in E-cadherin and Z01 were found. A successful restoration of all the abovementioned biomarkers’ levels was observed in group IV. Phytomedicine extracts (Urox^®^) were found to be potent in reversing RA-induced changes in several cystometric and biochemical parameters that are determinants of overactive bladder (OAB). The actions of Urox^®^ were proved to be dependent on several factors, such as growth factors and several OAB biomarkers but not pro-inflammatory cytokines.

## Introduction

Antimuscarinic drugs are well-known agents that have earned their place in the nowadays management of overactive bladder (OAB). One should not forget about their dark side, i.e., side effects leading to the phenomenon of the low proportion of patients still on drugs at 1-year observation (Lua et al., 2017). According to the authors of the National Overactive Bladder Evaluation (NOBLE) study, OAB exerts a clinically significant impact on quality of life, such as sleep, and other realms of mental health (Stewart et al., 2003). Thus, having in mind that OAB is a debilitating condition with a great and long-term impact on social activity, it seems natural that new substances receive growing attention (Chughtai et al., 2013). It is coherent with the observation that nearly 75% of individuals with OAB reach for complementary medicine (Chughtai et al., 2013).

The incorporation of herbal drugs into the current management of OAB has been shown to be an ambiguous attempt though (Chughtai et al., 2013). The state-of-the-art summary is that no competitive agents to the existing pharmacological treatment have been found and even promising candidates lack well-designed studies to confirm their efficacy (Zhou et al., 2019). Among several herbal agents for OAB, the most studied were Gosha-jinki-gan (Kajiwara and Mutaguchi, 2008), Hachi-mi-jio-gan (Ito et al., 2009), resiniferatoxin, or capsaicin (Heng et al., 2011). Little has been done in phytomedicine for OAB since the last decade though (Betschart et al., 2013; Chughtai et al., 2013). In general, herbal drugs were reported to have a lower efficacy but definitely fewer adverse effects than cholinolytics (Xiao et al., 2016) and have been perceived as rather complementary treatment than monotherapy (Xiao et al., 2016; Chen et al., 2021). The results of few clinical trials have been published, e.g., Betschart et al. (2013) analyzed *Bryophyllum pinnatum* for its safety and effectiveness, Xiao et al. (2016) focused on *Weng-li-tong*, Chen et al. (2021) used cinnamon patch for alleviating OAB symptoms, and finally, Schloss et al. (2019, 2021) considered Urox^®^ for the treatment of nocturnal enuresis and Schoendorfer et al. (2018) for alleviating OAB symptoms and urinary incontinence. The latter is a herbal mixture of *Crateva nurvala*, *Equisetum arvense*, and *Lindera aggregata* that has been recognized in traditional medicines for years (Schoendorfer et al., 2018). Since 2011, Urox^®^ has been also enlisted in the Therapeutic Goods Administration in Australia, with below 0.1% of adverse events and no reports on interference with known medications (Schoendorfer et al., 2018). The true nature of the mechanisms involved in its potential efficacy in OAB remains undetermined though.

In our previous studies, we had confirmed that detrusor overactivity was induced by retinyl acetate (RA) in rats (Wróbel et al., 2015; Wróbel et al., 2020) and described that *Potentilla chinensis* extract (PCE) has the potential to reverse RA–induced detrusor overactivity (DO) (Wróbel et al., 2020). The aim of the current study was to investigate whether phytomedicine extracts from *Lindera aggregata* root, *Equisetum arvense* stem, and *Crateva nurvala* stem bark (Urox^®^) would reverse RA-induced changes in several cystometric and biochemical parameters characteristic of bladder overactivity and to thus check if this herbal supplement could be a reasonable strategy, as a future pharmacological treatment for patients with OAB.

## Materials and Methods

All applied experimental procedures were approved by the local ethics committee and were conducted in accordance with the European Communities Council Directive of 22 September 2010 (2010/63/EU).

### Animals Involved in the Experiments

There were 60 female Wistar rats (weighted initially 250 g) involved in the experiments. The animals were located individually in environmentally controlled rooms (temperature of 22–23°C, natural light/dark cycle, relative humidity ∼45–55%), i.e., in the metabolic cages (3700M071, Tecniplast, West Chester, PA, United States) with unlimited access to water and food. The rats were randomly assigned to the four experimental groups consisting of 15 animals each. The experimental groups received saline (the control group, CON), only retinyl acetate (RA), Urox^®^ (UROX; single dose as of 840 mg/day), and finally, the combination of retinyl acetate plus Urox^®^ (RA + UROX).

### Drugs

In the experiments, we used retinyl acetate (RA) (Sigma–Aldrich) and Urox^®^ (Seipel Group, Brisbane, Australia). RA was diluted to 0.75% solution with a mixture of Polysorbate 80 and saline. It was administered one time for 5 min at room temperature in the form of intravesical instillation for bladder detrusor overactivity induction, as described previously (Wróbel et al., 2015).

Urox^®^ was administered by oral gavage at a single daily dose of 840 mg for the consecutive 14 days. The dose of the administered agent was established based on the results of our previous preliminary studies (data not published). The control animals received a volume-matched dose of vehicle.

### Surgical Procedures

All the surgical procedures were performed as described previously (Wróbel et al., 2015; Wróbel and Rechberger, 2017). Briefly, rats were placed supine on a warming mattress (37°C). The animal bladders were catheterized with a polyethylene catheter, and directly after removing the residual urine, 0.75% RA solution (to induce detrusor overactivity) or vehicle was administered in the form of intravesical instillation and left for 5 minutes. After emptying the bladder from saline/RA, it was gently filled with saline for washing purposes, and then the catheter was removed. The abdominal wall was opened with a 10-mm midline incision. The urinary bladder was separated from the adjacent tissues and a double lumen polyethylene catheter (inside diameter, i.d., 0.28 and outside diameter, o.d., 0.61 mm; BD, Franklin Lakes, NJ, United States) was inserted through a small incision into the bladder dome and fixed with a 6-0 Vicryl suture. In the same session, the carotid artery was cannulated with a polyethylene catheter (i.d. 0.28 and o.d. 0.61 mm; BD) filled with 40 IU/ml heparinized physiological saline to measure the blood pressure. The catheters were tunneled subcutaneously and exteriorized in the retroscapular area, where they were connected with a plastic adapter, to avoid the risk of removal by the animal. Finally, Healon (Pharmacia AB) at a dose of 0.85 ml was applied around the urinary bladder to avoid adhesions. The abdomen was closed in multiple layers.

### Conscious Cystometry

Cystometric investigations were performed 16 days after the surgical procedures (i.e., 2 days after the last dose of Urox), as we have described elsewhere (Wróbel et al., 2019a; Wróbel et al., 2019b). The bladder catheter was connected *via* a three-way stopcock to a pressure transducer (FT03; Grass Instruments) situated at the level of the bladder and to a microinjection pump (CMA 100; Microject, Solna, Sweden) for recording intravesical pressure and for infusing physiological saline into the bladder. Conscious cystometry was performed at a constant infusion rate of 0.05 ml/min, i.e., 3 ml/h at room temperature (22°C) to elicit repetitive voiding. The analog signal obtained from the pressure transducer was amplified and digitized using the Polyview system (Grass Instruments). Micturition volumes were measured by means of a fluid collector attached to a force displacement transducer (FT03C; Grass Instruments). Both transducers were connected to a polygraph (7 DAG; Grass Instruments). Cystometry profiles and micturition volumes were recorded continuously on a Grass polygraph (Model 7E; Grass Instruments) and were determined graphically. The data were analyzed using a sampling rate of 10 samples/s. The measurements in each animal represent the average of five bladder micturition cycles after obtaining repetitive voiding. The mean values from all animals in each condition were averaged to create pooled data for each condition. The cystometric parameters were defined previously (Wróbel et al., 2015; Wróbel et al., 2020). All procedures were performed by a person blinded to the treatments.

### Urothelium Thickness Measurement

The measurement of urothelium thickness was carried out as we have described before (Wróbel et al., 2019a). The image analyzer computer system Leica QWin 500 Image Analyzer (Leica Imaging Systems Ltd., Cambridge, England) was used to evaluate the urothelium thickness in micrometers using the interactive measure menu and hematoxylin and eosin–stained sections. A mean of 15 readings was estimated from 5 serial sections from slides of each animal in each group using low magnification (×10).

### Bladder Blood Flow

The bladder blood flow (BBF) was determined using a laser Doppler blood perfusion imager (PeriScan PIM 3, Perimed) and presented as changes in the laser Doppler frequency in a color scale. The BBF was measured five times for each rat bladder immediately after bladder emptying.

### Assessment of Cardiovascular Parameters and Diuresis

After completing cystometry, the animals were placed individually in metabolic cages for 24 h in order to evaluate the effects of Urox on the mean arterial pressure (MAP), heart rate (HR), and daily urine production (UP), as described previously (Wróbel et al., 2015).

### Determining the Expression Levels of c-Fos in Central Micturition Areas

c-Fos expression was determined in the central micturition areas: medial preoptic area (MPA), pontine micturition center (PMC), and the ventrolateral periaqueductal gray (vlPAG). Based on the findings from the stereotactic atlas of the rat’s brain and the bregma as the point of reference, the PMC, vlPAG, and MPA were isolated, as described by Kim et al. (2012). Ten sections on average per region were obtained from each rat. The PMC was denoted as the region spanning bregma −9.68 to −9.80 mm; vlPAG was the region spanning bregma −7.64 to −8.00 mm; the MPA was the region spanning from bregma −0.26 to 0.80 mm. Next, after homogenizations, the respective concentrations were determined using a high-sensitivity commercial immunoenzymatic assay in line with the manufacturer’s instructions (c-Fos; MyBioSource, MBS729725), as described previously (Wróbel et al., 2018).

### Biochemical Analyses

Based on ELISA experiments, the levels of the following biomarkers were determined in the bladder urothelium: Calcitonin Gene-Related Peptide (CGRP; Biomatik, CN EKU02858), Organic Cation Transporter 3 (OCT3; antibodies-online, CN ABIN6227163), Transient Receptor Potential Cation Channel, Subfamily V, Member 1 (TRPV1; LSBio, LS-F36019), E-Cadherin (CDH1; Abbexa Ltd., abx052816), Tight Junction Protein 1 (ZO1; Cusabio, CSB-E17287-r), ATP Citrate Lyase (ATP; LifeSpan BioSciences, LS-F10730), IL-1β (Cloud-Clone; SEA563Ra), IL6 (LifeSpan BioSciences; LS-F25921-1), TNF-α (LifeSpan BioSciences; LS-F5193), Malondialdehyde (Biomatik, CN EKF57996), 3-nitrotyrosine (LifeSpan BioSciences; CN LS-F40120-1). The respective levels of vesicular acetylcholine transporter (VAChT; LifeSpan BioSciences, CN LS-F12924-1) and Rho Kinase (ROCK1; LifeSpan BioSciences, LS-F32208) were examined in the bladder detrusor muscle, whereas the concentration of nerve growth factor (NGF; LifeSpan BioSciences, CN LS-F25946-1) and brain-derived neurotrophic factor (BDNF; PROMEGA, CN G7610) were determined in the urine. All measurements were carried out according to the manufacturers’ instructions. Each sample was measured in duplicate. The results are presented in picograms/milliliters.

### Study Design

After performing the surgical procedures, the Urox or vehicle was administered by oral gavage for 14 days. The cystometric investigation and bladder blood flow (BBF) assessment were performed 2 days after the last dose of Urox. Then, the rats were put into the metabolic cages (3700M071, Tecniplast) for 24 h to assess UP, HR, and MAP. Next, the animals were killed by decapitation, and urothelium thickness measurement and biochemical analyses were performed.

### Statistical Analysis

The obtained data were assessed by the two-way analysis of variance (ANOVA) followed by a Bonferroni *post hoc* test (Statistica, v. 10, StatSoft, Inc., Tulsa, OK, United States). All results are presented as the mean ± standard error of the mean (SEM). *p* < 0.05 was considered statistically significant with 95% confidence.

## Results

### Effects of Urox^®^ on Retinyl Acetate–Induced Changes in Cystometric Parameters

As would be expected from an agent that stimulated a response similar to OAB, RA initiated changes in the cystometric parameters in both the storage and voiding phase (please refer to Table 1). In the storage phase, the following parameters were improved: basal pressure (BP), threshold pressure (TP), detrusor overactivity index (DOI), non-voiding contraction frequency (FNVC), volume threshold to elicit NVC (VTNC), non-voiding contraction amplitude (ANVC), and bladder compliance (BC) (Table1). In the voiding phase, the voided volume (VV), area under the pressure curve (AUC), and intercontraction interval (ICI) were increased, being all suggestive of symptomatic OAB. Importantly, in rats that received the combination of RA and Urox, reversed changes were observed. Firstly, a decrease in the storage phase parameters (BP, DOI, FNVC, and ANVC) was noted in contrast to the RA group. Furthermore, in the combination group, other voiding (VV, AUC, ICI) and storage phase (TP, VTNC, and BC) parameters were increased, being indicative of reversing symptoms induced by RA.

**TABLE 1:** The effects of UROX^®^ on retinyl acetate (RA)–induced changes in cystometric parameters.

	Control	Retinyl acetate	Urox^®^	Retinyl acetate + Urox^®^
Storage phase				
Basal pressure (BP, cm H_2_O)	2.5 ± 0.61	4.2 ± 0.72 ****	2.2 ± 0.45 ^^^^	3 ± 0.9 ^^^^
Threshold pressure (TP, cm H_2_O)	7.3 ± 1.1	4.1 ± 0.88 ****	6.5 ± 1.2 ^^^^	7.5 ± 1.6 ^^^^
Detrusor overactivity index (DOI, cm H_2_O/ml)	49 ± 13	173 ± 54 ****	55 ± 24 ^^^^	94 ± 17 ^^^^, **
Non-voiding contractions frequency (FNVC, times/filling phase)	0.36 ± 0.18	3.8 ± 1.7 ****	0.44 ± 0.27 ^^^^	2.3 ± 0.92 ^^^
Volume threshold to elicit NVC (VTNC, %)	69 ± 13	47 ± 12 ***	66 ± 14 ^^	65 ± 15 ^^
Non-voiding contractions amplitude (ANVC, cm H_2_O)	2.4 ± 0.31	5.8 ± 2 ****	2.4 ± 0.24 ^^^^	3.4 ± 0.89 ^^^^
Bladder compliance (BC, ml/cm H_2_O)	0.28 ± 0.063	0.15 ± 0.020 ****	0.24 ± 0.051 ^^^^	0.22 ± 0.044 ^^^
Voiding phase				
Micturition voiding pressure (MVP, cm H_2_O)	37 ± 6.9	31 ± 7	36 ± 9.3	36 ± 7.4
Intercontraction interval (ICI, s)	1,080 ± 186	720 ± 152 ****	1,056 ± 204 ^^^^	890 ± 150 ^
Voided volume (VV, ml)	0.76 ± 0.14	0.57 ± 0.15 *	0.75 ± 0.15 ^	0.76 ± 0.23 ^
Post-void residual (PVR, ml)	0.067 ± 0.017	0.069 ± 0.015	0.067 ± 0.021	0.081 ± 0.013
Area under the pressure curve (AUC, cm H_2_O/sec)	12 ± 2.7	19 ± 2.8 ****	13 ± 2.5 ^	14 ± 2.6 ^

Values are expressed as mean ± SEM. * or ^ *p* < 0.05; ** or ^^ *p* < 0.01; *** or ^^^ *p* < 0.001, **** or ^^^^*p* < 0.0001. *Significantly different from the control group. ^ Significantly different from the RA group. One-way ANOVA: for BP F(3.56) = 24, *p* < 0.0001; for TP F(3,56) = 25, *p* < 0.0001; for VV (F(3,56) = 4.5, *p* < 0.01; for ICI F(3,56) = 14, *p* < 0.0001; for DOI F(3,56) = 50, *p* < 0.0001; for FNVC F(3,56) = 43, *p* < 0.0001; for VTNVC F(3,56) = 8, *p* < 0.0001; for ANVC F(3,56) = 30, *p* < 0.0001; for BC F(3,56) = 21, *p* < 0.0001; and for AUC F(3,56) = 22, *p* < 0.0001. BP, basal pressure (cm H_2_O), TP, threshold pressure (cm H_2_O), MVP, micturition voiding pressure (cm H_2_O), VV, voided volume (ml), PVR, post-void residual (ml), ICI, intercontraction interval (s), DOI, detrusor overactivity index (cm H_2_O/ml), FNVC, non-voiding contractions frequency (times/filling phase), VTNVC, volume threshold to elicit NVC (%), ANVC, non-voiding contractions amplitude (cm H_2_O), BC, bladder compliance (ml/cm H_2_O), AUC, the area under the pressure curve (cm H_2_O/sec).

### Effects of Urox^®^ on Retinyl Acetate–Induced Changes in Urothelium Thickness and Bladder Blood Flow

No statistically significant changes in the bladder epithelial thickness were observed in the respective experiments (Table 2). Similarly, no statistical significance was noted when bladder blood flow was analyzed both in RA and in RA plus Urox^®^ treatment groups.

**TABLE 2:** The effects of UROX^®^ (840 mg p.o., once daily for 14 consecutive days) on retinyl acetate (RA)–induced changes in urothelium thickness and bladder blood flow.

	Control	Retinyl acetate	Urox^®^	Retinyl acetate + Urox^®^
Urothelium thickness	56 ± 9.8	58 ± 12	53 ± 8.1	61 ± 9.8
Bladder blood flow	132 ± 13	126 ± 17	135 ± 12	133 ± 15

Values are expressed as mean ± SEM; no statistically significant changes were observed.

### Effects of Urox^®^ on Retinyl Acetate–Induced Changes in Cardiovascular Parameters and Diuresis

In the present experiments, we failed to notice any significance for both basic cardiovascular (MAP and HR) parameters and urine volume (please see Table 3).

**TABLE 3:** The effects of the UROX^®^ on retinyl acetate (RA)–induced changes in cardiovascular parameters and diuresis.

	Control	Retinyl acetate	Urox^®^	Retinyl acetate + Urox^®^
Mean arterial pressure (MAP, mm Hg)	114 ± 11	122 ± 16	110 ± 15	118 ± 14
Heart Rate (HR, beats/min)	221 ± 24	207 ± 21	211 ± 17	222 ± 22
Urine Production, (UP, ml/day)	20 ± 1.5	19 ± 2.7	20 ± 2.8	21 ± 3.2

Values are expressed as the mean ± SEM; no statistically significant changes were observed.

### Effects of Urox^®^ on Retinyl Acetate–Induced Changes in the Expression Levels of c-Fos in Central Micturition Areas

**FIGURE 1:**
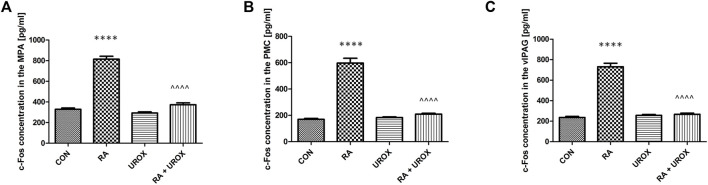
The effects of Urox^®^ (840 mg p.o., once daily for 14 consecutive days) on c-Fos expressions in the neuronal voiding centers: **(A)** medial preoptic nucleus (MPA), **(B)** pontine micturition center (PMC), and **(C)** ventrolateral periaqueductal gray (vlPAG) after the induction of overactive bladder with retinyl acetate (RA). Concentration of c-Fos in each group (pg/ml): control group (CON), RA group (RA), Urox-only group (UROX) and RA group treated with UROX (RA + UROX). Values are expressed as mean ± SEM.**** or ^^^^*p* < 0.0001; *significantly different from the control group; ^significantly different from the RA group. One-way ANOVA: for MPA: F (3.56) = 163, *p* < 0.0001; for PMC: F (3.56) = 115, *p* < 0.0001; for vlPAG: F (3.56) = 155, *p* < 0.0001.

As presented in Figure 1, there were significant changes in c-Fos expression after a single administration of RA in all the analyzed compartments when compared to the control group. On the contrary, Urox^®^ demonstrated a potential to significantly lower the expression of c-Fos after RA exposure (RA plus Urox^®^ group compared to RA group) in all three studied micturition areas (MPA, PMC, and vlPAG).

### Effects of Urox^®^ on Retinyl Acetate–Induced Changes in Biochemical Analyses of Biomarkers

**FIGURE 2:**
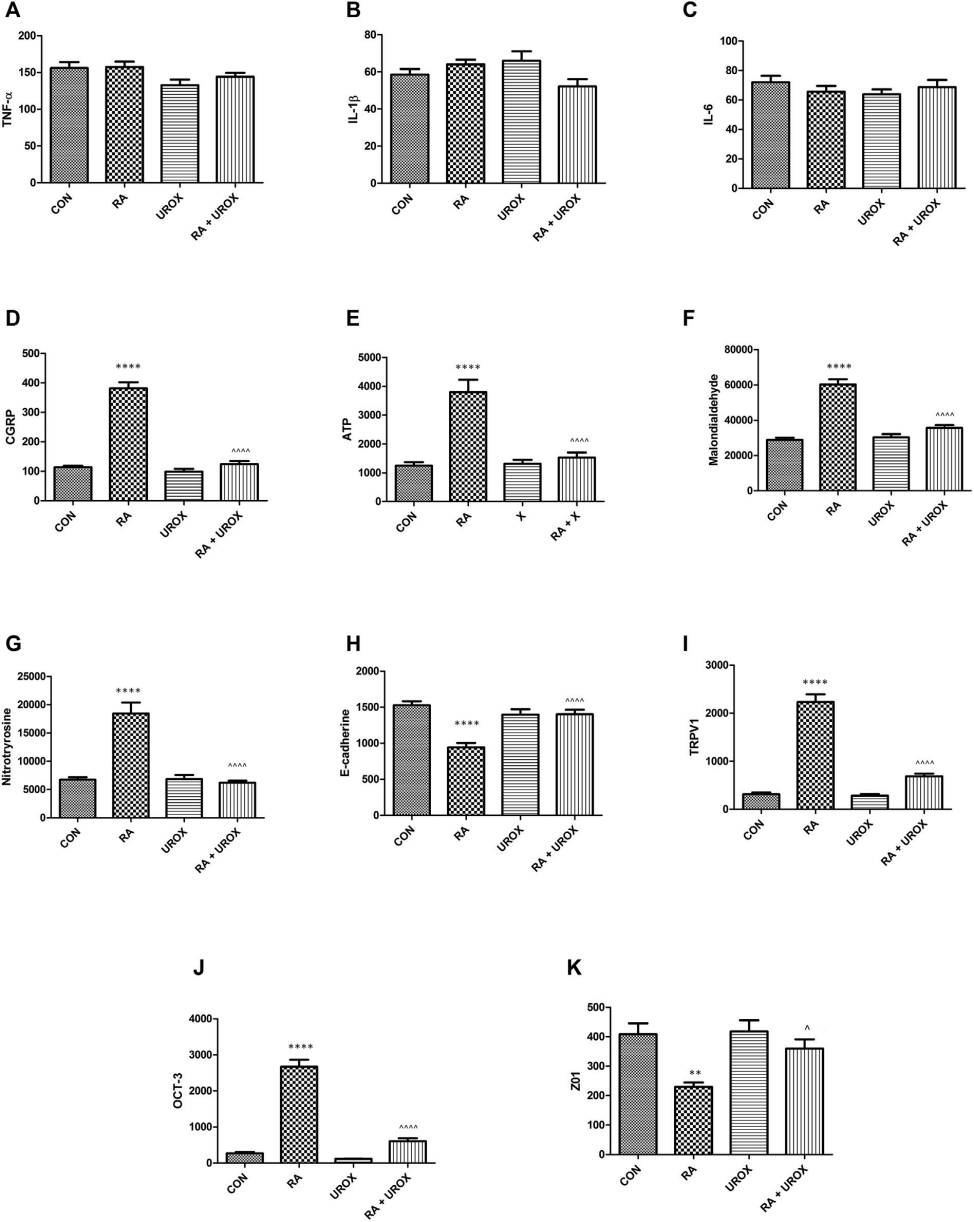
The influence of the 14-day administration of Urox^®^ (840 mg p.o.) on biomarkers’ level (pg/ml) in the bladder urothelium: **(A)** TNF-α, **(B)** IL-1β, **(C)** IL-6, **(D)** CGRP, **(E)** ATP, **(F)** malondialdehyde, **(G)** 3-nitrotyrosine, **(H)** E-cadherin, **(I)** TRPV1, **(J)** OCT-3, and **(K)** Z01 in rats subjected to a single injection of retinyl acetate (RA). Values are expressed as mean ± SEM. **p < 0.01, ****p < 0.001 *versus* saline, ^*p* < 0.05, ^^^^*p* < 0.0001 *versus* RA (*n* = 15 rats per group). One-way ANOVA: for CGRP: F(3.56) = 115, *p* < 0.0001; for ATP: F(3.56) = 24, *p* < 0.0001; for malondialdehyde: F(3.56) = 51, *p* < 0.0001; for 3-nitrotyrosine: F(3.56) = 31, *p* < 0.0001; for E-cadherin: F(3.56) = 16, *p* < 0.0001; for TRPV1: F(3.56) = 113, *p* < 0.0001; for OCT-3: F(3.56) = 122, *p* < 0.0001; and for Z01: F(3.56) = 7.5, *p* < 0.0001. CON, control.

**FIGURE 3:**
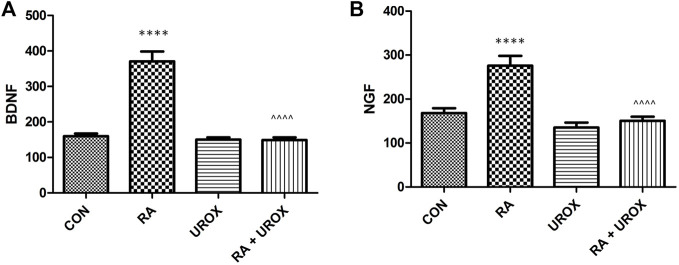
The influence of the 14-day administration of Urox^®^ (840 mg p.o.) on biomarkers’ level (pg/ml) in urine: **(A)** BDNF and **(B)** NGF in rats subjected to a single injection of retinyl acetate (RA). Values are expressed as mean ± SEM. ****p < 0.001 *versus* saline, ^^^^*p* < 0.0001 *versus* RA (*n* = 15 rats per group). One-way ANOVA: for BDNF: F(3.56) = 52, *p* < 0.0001; and for NGF: F(3.56) = 19, *p* < 0.0001; CON, control.

**FIGURE 4:**
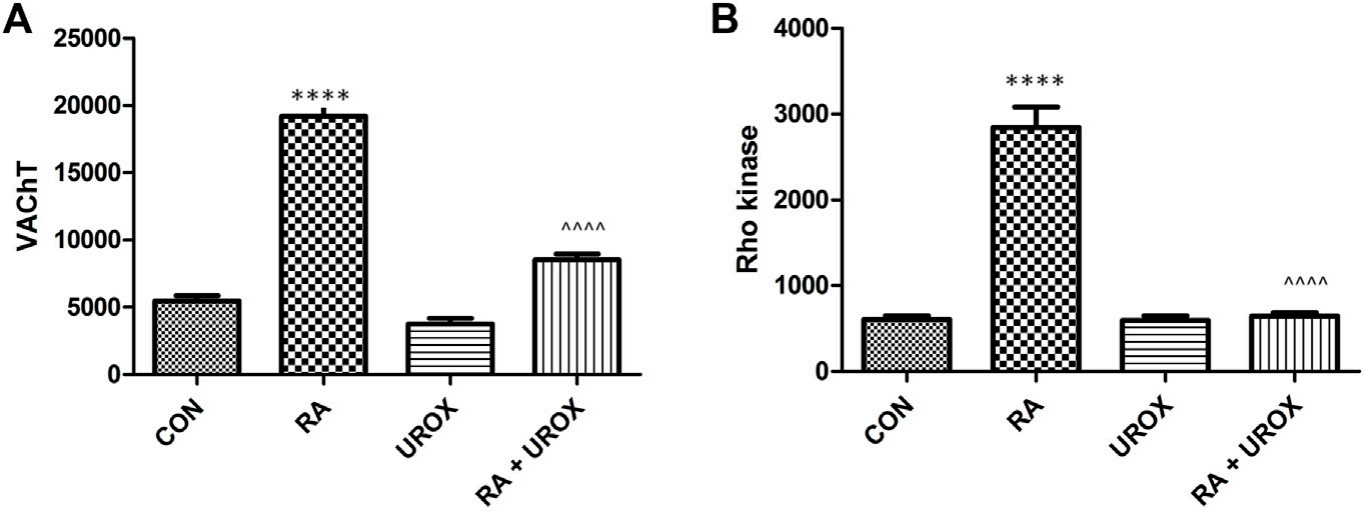
The influence of the 14-day administration of Urox^®^ (840 mg p.o.) on biomarkers’ level (pg/ml) in the bladder detruso r muscle: **(A)** vesicular acetylcholine transporter (VAChT) and **(B)** Rho kinase. Values are expressed as the mean ± SEM. ****p < 0.001 *versus* saline, ^^^^*p* < 0.0001 *versus* RA (*n* = 15 rats per group). One-way ANOVA: for VAChT: F(3.56) = 97, *p* < 0.0001 and for Rho kinase: F(3.56) = 80, *p* < 0.0001. CON, control.

No significant changes in the levels of the analyzed biomarkers after administration of Urox^®^ only were found, when collated with the saline-treated group (Figures 2–4). On the other hand, RA instillation resulted in an elevation of several biomarkers found in bladder urothelium (CGRP, ATP, malondialdehyde, 3-nitrotyrosine, TRPV1, and OCT-3), while a decrease in permeability markers (E-cadherin and Z01) was noted (please see Figure 2). Interestingly, no changes were found in the case of pro-inflammatory cytokines—TNF-α, IL-1β, and IL-6 (Figure 2). As for biomarkers found in urine, BDNF and NGF were increased (please refer to Figure 3). Finally, VAChT and Rho kinase that were assessed in bladder detrusor muscle were elevated after RA installation (Figure 4). Definitely, we aimed at successful restoration of all the abovementioned changes in the biomarkers’ level after administration of Urox^®^ (RA + UROX combination group). As it was presented in Figures 2–4, all the biomarkers that were abnormally elevated due to the presence of RA were normalized after installation of Urox^®^, which stands in line with our hypothesis that the drug may reverse some of the OAB biochemical determinants.

## Discussion

In the present study, we shed some light on the potential mechanisms involved in OAB symptoms reduction after Urox^®^ administration. Firstly, in the established, RA-induced model of OAB, we observed typical urodynamic parameters, indicative of increased voiding frequency, with crucial changes in the central micturition areas for c-Fos expression and shifts in the levels of biomarkers detected in the bladder urothelium/detrusor muscle or urine. Then, the abovementioned phenomena were successfully reversed due to the 14-day oral administration of a herbal drug, Urox^®^.

Previous studies have revealed the potency of Urox^®^ components in alleviating symptoms of OAB. A randomized double-blind placebo-controlled trial demonstrated that Urox^®^ reduces the frequency of urinary per day and episodes of nocturia, and alleviates urgency and total incontinence with no severe adverse events reported (Schoendorfer et al., 2018). Schauss and Spiller (2006) reported that UroLogic™ a herbal drug containing *Equisetum arvense* and *Crateva nurvala*, was effective in OAB symptoms reduction, together with inducing relief for bladder discomfort. Furthermore, in another study, they found no interference with P450 cytochromes, which is indicative of its safety when being used with other medications (Schauss and Elizabeth, 2006). In turn, *Lindera aggregata* was used for frequent voiding or incontinence (Schoendorfer et al., 2018). Based on the available clinical studies (the dosages between 420 and 840 mg, administered for 2–6 weeks), we included 840 mg of Urox^®^ for 14 consecutive days (Ito et al., 2009; Heng et al., 2011). What is more, we confirmed the value in pretests (data not presented) as a minimally effective dose with no behavioral changes in rats, including rat activity.

Recently, we established a novel model of OAB using RA-induced changes in rat bladder, in which cystometric parameters evoked by RA were reversed by the administration of oxybutynin chloride (Wróbel et al., 2015). In our present experiments, we observed attenuation of the changes caused by RA after administration of Urox^®^. The most significant storage phase parameters were improved, i.e., BP, DOI, FNVC, and ANVC were lowered, while TP, VTNC, and BC were increased. These parameters greatly resemble the most important features of OAB to be observed in the urodynamic study. This leads to the conclusion that Urox^®^ is potent in resolving OAB symptoms in our model. The normalization of the storage phase parameters may be due to the desensitization of the afferent fibers reaching the urinary bladder, as described previously (Wróbel et al., 2020). What is worth emphasizing is that no influence on MVP and PVR were observed, which suggests that Urox^®^ does not compromise normal voiding. The direct proof for the action of Urox^®^ on detrusor stability was the ability to lower DOI, as traditionally in *in vivo* studies, unstable detrusor contractions appear in the urine storage period as a standard finding to diagnose OAB (Pan et al., 2012).

Here, we focused on determining the mechanisms that may play a role in Urox^®^ action. We observed no influence on bladder epithelial thickness both in the RA-induced DO model and Urox^®^ or RA plus Urox^®^ groups. Furthermore, we did not recognize any statistically important changes in bladder blood supply. Blood flow in the bladder is not well understood (Andersson et al., 2017). Andersson et al. (1985) found in an animal model that vasodilation increased with bladder capacity and was lowered in an empty bladder. However, some authors indicate that there is a decrease in mucosal rather than muscle layer blood supply during the filling phase (Andersson et al., 2017). Still, our experiments did not indicate any connection with both OAB pathophysiology and the possible effect of Urox^®^ on these parameters.

When focusing on the safety of the drug, it should be emphasized that no cardiovascular effects were noted, including changes in blood pressure or heart rate in normal rats. Furthermore, no diuretic effect was observed as the urine output remained the same after Urox^®^ administration. These observations should be confirmed in human studies but may be allowed to remain optimistic as for the administration of the drug in patients with cardiovascular diseases. On the other hand, the elevated urine production would impose detrusor instability and exacerbate the filling-phase lower urinary tract symptoms.

Most importantly, in the present study, we performed experiments to assess the effects of Urox^®^ on c-Fos expression in the neuronal voiding centers (MPA, PMC, and vlPAG). c-Fos expression is thought to be a marker of neuronal activity (Lee et al., 2003), which becomes highly increased due to bladder sensation in PAG and PMC (Dinis et al., 2004). The central micturition regions are activated by bladder stimuli in OAB, which in turn leads to an increase of c-Fos (Kim et al., 2012). Kim et al. (2012) reported that the expressions of c-Fos in the neuronal voiding centers were significantly elevated in the cyclophosphamide-induced OAB model. Here, we have found that RA induced significant c-Fos expression in all the analyzed centers. Additionally, the effect was attenuated by Urox^®^. Thus, the lowered levels of c-Fos may be a significant sign announcing symptoms relief due to OAB and may partly explain one of the possible mechanisms of Urox^®^ activity.

In biochemical analyses, we also found that Urox^®^ did not reveal any activity for the induction of the proinflammatory cytokines (TNF-α, IL-1β, and IL-6). In our model of DO induction based on RA, no such mechanisms were determined to play a role, as well. Furthermore, urinary BDNF and NGF were examined as potential biomarkers for overactive bladder (OAB) (Wang et al., 2014). Additionally, the NGF levels were positively correlated with the severity of OAB (Tian et al., 2019). In our experiments, these were urine BDNF and NGF levels that were elevated in the RA group and normalized in the combination group due to the positive effect of Urox^®^. Another novel biomarker in OAB diagnostics is malondialdehyde (Rada et al., 2020), and increased values of this substance together with the concentration of 3-nitrotyrosine were detected both in OAB patients and *in vivo* studies on OAB and bladder inflammation (Kim et al., 2012; Barut et al., 2019; Tian et al., 2019). Similar to the abovementioned growth factors, we observed a significant fall in both malondialdehyde and 3-nitrotyrosine levels in the combination group when compared to RA-treated animals.

Increased density of suburothelial nerve fibers in OAB individuals that are immunoreactive for CGRP are reported (Fowler et al., 2008). On the other hand, onabotulinumtoxinA is thought to be successful in the treatment of OAB due to the inhibition of CGPR release in the bladder (Rapp et al., 2006). In our experiments, we proved that similar mechanisms can be involved in the efficacy of Urox^®^ in the animal model of OAB. Furthermore, the expression of multipotent enzymes that play a role at various steps of molecular metabolism, i.e., ATP and Rho kinase, were also elevated due to RA action. The possible multifactorial mechanisms of Urox^®^ may be the reason for its potency to induce normalization of their levels in our experiments.

An increased expression of TRPV1 in bladder mucosa of OAB patients was proved to be directly correlated to OAB occurrence (Zhang et al., 2015). Additionally, there is a significant elevation in molecules responsible for acetylcholine transportation: VAChT and OCT-3 in patients with OAB (Wolf-Johnston et al., 2012). All these possible additional molecular pathological pathways may be attenuated with Urox^®^, as we have shown above. The decreased levels of VAChT in detrusor after Urox^®^ treatment restrict detrusor muscle contractility which corresponds with improved cystometric results described in the respective sections.

The increased permeability of urothelium lies at the origins of bladder inflammation (Hurst et al., 2015). Here, we included Z01 and E-cadherin in the experiments to investigate this idea and revealed that again Urox^®^ increased the expression of these two biomarkers. It strongly suggests that Urox^®^ limits the pathological sensitization of the neurons located beneath the urothelium. As a consequence, we strongly suggest the incorporation of several biomarkers of OAB into clinical practice as only future studies may reveal the optimal set.

We are aware of some of the limitations of our study. We included only female rats, and it can lead to potential bias in the interpretation of some of the results. It involves the necessity for further experiments in male rats. The properties of Urox^®^ found *in vitro* and *in vivo* cannot be directly generalized and is expected in unchanged form in the human population, which entails clinical studies. Both dosage and treatment period were selected based on preliminary unpublished data as no exact analyses are available in the literature.

## Conclusion

In conclusion, phytomedicine extracts (Urox^®^) were found to be potent to reverse RA-induced changes in both several cystometric and biochemical parameters that are determinants of OAB. We observed no effects on basic cardiovascular parameters and daily urine output, which may promote the initiation of the analysis of its safety in humans. The actions of Urox^®^ were proved to be dependent on several factors, such as growth factors and several OAB biomarkers but not pro-inflammatory cytokines.

## Data Availability

The raw data supporting the conclusion of this article will be made available by the authors, without undue reservation.
